# *Six2* Is a Coordinator of LiCl-Induced Cell Proliferation and Apoptosis

**DOI:** 10.3390/ijms17091504

**Published:** 2016-09-08

**Authors:** Jianing Liu, Pan Ju, Yuru Zhou, Ya Zhao, Yajun Xie, Yaoshui Long, Yuping Gu, Dongsheng Ni, Zhongshi Lyv, Zhaomin Mao, Jin Hao, Yiman Li, Qianya Wan, Quist Kanyomse, Yamin Liu, Yue Xiang, Ruoli Wang, Xiangling Chen, Junman Zhang, Xihan Liu, Hui Zhao, Qin Zhou, Ge Li

**Affiliations:** 1 Division of Molecular Nephrology and the Creative Training Center for Undergraduates, the Ministry of Education Key Laboratory of Clinical Diagnostics, School of Laboratory Medicine, Chongqing Medical University, Chongqing 400016, China; liujianingb@gmail.com (J.L.); 18883936591@163.com (P.J.); zhouyuru93@gmail.com (Yu.Z.); xianzhaoya@gmail.com (Ya.Z.); yjxie@genetics.ac.cn (Y.X.); longyaoshui@gmail.com (Ya.L.); littlebottlesky@gmail.com (Y.G.); dongshengni@outlook.com (D.N.); zhongshilyu@gmail.com (Z.L.); maozhaomin8@gmail.com (Z.M.); lanyxiu@163.com (J.H.); liyimanb@gmail.com (Yi.L.); qy.wan@Outlook.com (Q.W.); quistmansa@gmail.com (Q.K.); liuyamin2013@126.com (Yam.L.); zhouqin@cqmu.edu.cn (Q.Z.); 2 The fifth Clinical College of Medicine, Chongqing Medical University, Chongqing 400016, China; 3 Department of Laboratory Medicine, the First Hospital of Xi’an, Xi’an 710002, China; 4 Department of scientific and technological activity, Chongqing Yucai Middle School, Chongqing 400016, China; xiangyue990806@foxmail.com (Y.X.); puzimei@foxmail.com (R.W.); winster.ling@foxmail.com (X.C.); zjm15736451573@foxmail.com (J.Z.); elther@foxmail.com (X.L.); 5 Key Laboratory for Regenerative Medicine, Ministry of Education, School of Biomedical Sciences, Faculty of Medicine, The Chinese University of Hong Kong, Hong Kong, China; zhaohui@cuhk.edu.hk; 6 The Center of Experimental Teaching Management, Chongqing Medical University, Chongqing 400016, China

**Keywords:** LiCl, metanephric mesenchyme cells, cell proliferation, cell apoptosis, *Six2*

## Abstract

The metanephric mesenchyme (MM) cells are a subset of kidney progenitor cells and play an essential role in mesenchymal-epithelial transition (MET), the key step of nephron generation. *Six2*, a biological marker related to *Wnt* signaling pathway, promotes the proliferation, inhibits the apoptosis and maintains the un-differentiation of MM cells. Besides, LiCl is an activator of *Wnt* signaling pathway. However, the role of LiCl in cellular regulation of MM cells remains unclear, and the relationship between LiCl and *Six2* in this process is also little known. Here, we performed EdU assay and flow cytometry assay to, respectively, detect the proliferation and apoptosis of MM cells treated with LiCl of increasing dosages. In addition, reverse transcription-PCR (RT-PCR) and Western-blot were conducted to measure the expression of *Six2* and some maker genes of *Wnt* and bone-morphogenetic-protein (BMP) signaling pathway. Furthermore, luciferase assay was also carried out to detect the transcriptional regulation of *Six2*. Then we found LiCl promoted MM cell proliferation at low-concentration (10, 20, 30, and 40 mM). The expression of *Six2* was dose-dependently increased in low-concentration (10, 20, 30, and 40 mM) at both mRNA and protein level. In addition, both of cell proliferation and *Six2* expression in MM cells declined when dosage reached high-concentration (50 mM). However, *Six2* knock-down converted the proliferation reduction at 50 mM. Furthermore, *Six2* deficiency increased the apoptosis of MM cells, compared with negative control cells at relative LiCl concentration. However, the abnormal rise of apoptosis at 30 mM of LiCl concentration implies that it might be the reduction of GSK3β that increased cell apoptosis. Together, these demonstrate that LiCl can induce the proliferation and apoptosis of MM cells coordinating with *Six2*.

## 1. Introduction

During renal development, nephrons originate from a population of self-renewing *Six2* positive nephron progenitor cells, a part of metanephric mesenchyme (MM) cells [1,2,3]. Sine oculis homeobox homolog 2 (*Six2*), encoding a transcription factor, is required for the differentiation of MM cells, beginning with mesenchymal-to-epithelial transition (MET) to form early developing nephrons [4,5]. Furthermore, *Six2* regulates the proliferation (self-renewing) and consumption of nephron progenitor cells (a subset of MM cells) [1,6]. *Six2* promotes proliferation and inhibits apoptosis of MM cells to maintain MM cells in a progenitor state, which contributes to nephrogenesis [1,7]. Furthermore, in mouse kidney development, *Six2* deficiency promotes abnormal differentiation of mesenchyme cells and depletion of nephron progenitor cells in the cap mesenchyme (CM), finally leads to renal hypoplasia [1].

*Six2* is a crucial biomarker connected to *Wnt* signaling pathway that is highly conserved in evolution. *Wnt* signaling pathway functions in development by regulating numerous genes and proteins including *Six2* [8,9]. Most significantly, *Wnt*/*β-catenin* signaling determines cell fate of proliferation or differentiation in development [10]. Furthermore, lithium chloride (LiCl) is a classic activator of *Wnt* signaling by inhibiting GSK3β expression [11]. This lithium salt of hydrochloric acid is an important therapeutic agent and can regulate proliferation and apoptosis in cancer cells [12]. However, it is little known whether LiCl affects the proliferation and apoptosis of MM cells or not. Furthermore, the relationship between LiCl and *Six2* in the cellular regulation of MM cells is also unclear.

Here, we firstly demonstrated that LiCl can promote MM cells proliferation in low-concentration (10, 20, 30, and 40 mM). In mK3 cells, the expression of *Six2* and cell proliferation increased with dose-dependent of LiCl. Furthermore, knockdown of *Six2* can reduce the proliferation in LiCl-treated mK3 cells, showing that LiCl can induce the proliferation of mK3 cells via up-regulating *Six2* expression.

## 2. Results

### 2.1. LiCl Promotes the Proliferation of Metanephric mesenchyme (MM) Cells at Low-Concentration and Inhibits It at High-Concentration

**Figure 1:**
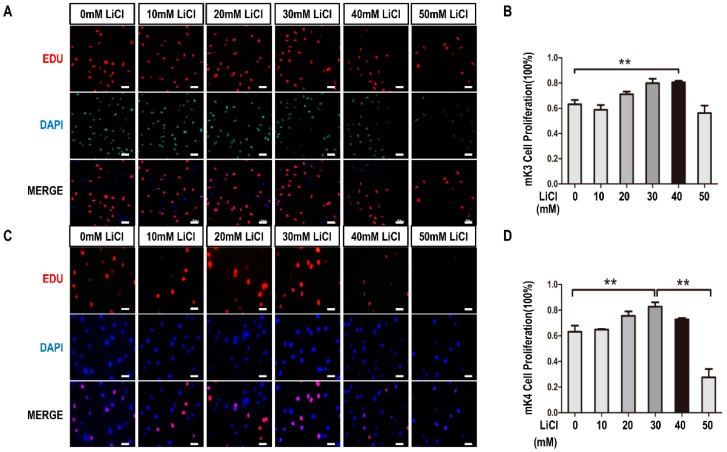
LiCl promotes cell proliferation in mK3 and mK4 cells. (**A**) mK3 cells were treated with LiCl of increasing dosages (0, 10, 20, 30, 40, and 50 mM) for 12 h and performed with 5-ethynyl-20-deoxyuridine (EdU) assays. Proliferating mK3 cells were labeled with EdU (red) and cell nucleuses were stained with DAPI (blue). The EdU results were accessed by fluorescent microscope (200×) with the scale bar representing 20 µm and the respective pictures were merged to the purple one; (**B**) Statistical analysis of mK3 cell proliferation. Values were presented as mean ± SEM (*n* = 3). *p*-values were calculated by Student *t*-test, ** *p* < 0.01 relative to control; (**C**) mK4 cells were treated in the same way as in (**A**) and the EdU assay were conducted; (**D**) Statistical analysis of mK4 cell proliferation. Values were presented as mean ± SEM (*n* = 3). *p*-values were calculated by Student *t*-test, ** *p* < 0.01 relative to control.

To clarify the relationship between LiCl and proliferation of MM cells, we treated the mK3 cells and mK4 cells with LiCl of increasing dosages (0, 10, 20, 30, 40, and 50 mM) and detected the proliferation rate using 5-ethynyl-2′-deoxyuridine (EdU) assay. The results Showed that mK3 cells proliferation rate was increased with concentration rising at low-concentration range (0, 10, 20, 30, and 40 mM) compared control cell, while it was partially reduced at high-concentration (50 mM) compared with the highest proliferation at 30 or 40 mM (Figure 1A,B). Similarly, in mK4 cells, cell proliferation rate was increased at low concentration of LiCl while the increasing was inhibited at 50 mM (Figure 1C,D). Therefore, we speculated that LiCl continuously promotes the proliferation of mK3 cells at low-concentration and inhibits it at high-concentration.

### 2.2. LiCl Up-Regulates the Expression of Six2 at Low-Concentration and Down-Regulates Six2 at High-Concentration

**Figure 2:**
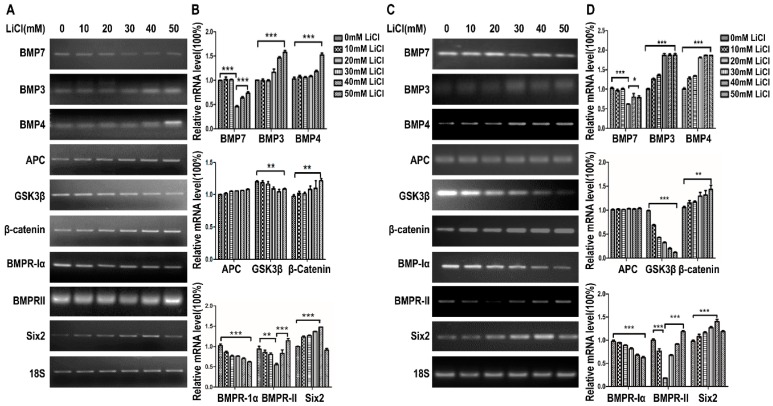
LiCl activates *Wnt* and BMP signaling pathway. (**A**) mK3 cells were treated with LiCl of increasing dosages (0, 10, 20 M, 30, 40, and 50 mM) for 12 h. The expression *Wnt* and BMP Signal markers were detected for confirming the function of LiCl by RT-PCR; (**B**) The relative mRNA expressions in mK3 cells was quantified by gray scan, normalized to the internal control 18S. Values were presented as mean ± SEM (*n* = 3). *p*-values were calculated by Student *t*-test, ** *p* < 0.01, *** *p* < 0.001 relative to control; (**C**) mK4 cells were treated same with mK3 cells and the mRNA expression of the same genes was tested by RT-PCR; (**D**) The relative mRNA expressions in mK4 cells were quantified by gray scan, normalized to the internal control 18S. Values were presented as mean ± SEM (*n* = 3). *p*-values were calculated by Student *t*-test, * *p* < 0.05, ** *p* < 0.01, *** *p* < 0.001 relative to control.

To demonstrate the relationship between LiCl and *Six2*, we isolated the total RNA from mK3 and mK4 cells treated with LiCl of increasing dosages and detected the expression of *Six2* and makers of *Wnt* and BMP signal pathway. As shown in Figure 2A,B, the expression of APC and *β-catenin* gene was increased gradually as concentration of LiCl rose, corresponding to the cell proliferation promotion, while the expression of GSK3Β was decreased (Figure 1B). Among BMP signal markers, the expression of BMP3 and BMP4 was increased, while the expression of BMP7 and BMPRII was reduced at low concentration of LiCl (0, 10, 20, and 30 mM) and then it was increased. The expression of BMPR-IA was reduced continually as concentration of LiCl increased. *Six2* expression is promoted at low dosages and the promotion was partially deleted at high dosage (50 mM), which was consistent with cell proliferation regulation trend as LiCl concentration rose (Figure 2A,B). All these detections and data were repeated in another cell line, mK4 cells (Figure 2C,D).

**Figure 3:**
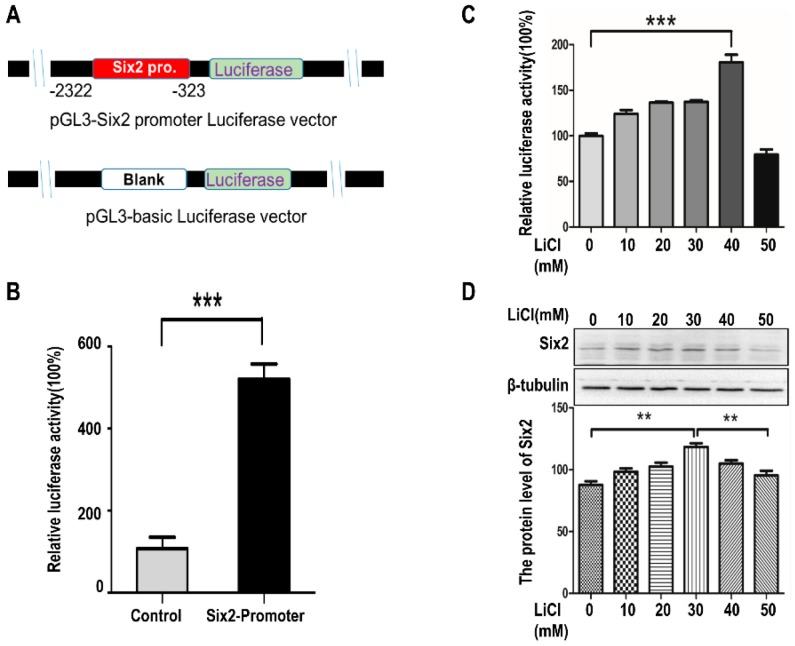
LiCl regulates the expression of *Six2* at mRNA and protein level. (**A**) pGL3-*Six2* promoter-Luciferase construction was simulate by diagram. The *Six2* promoter ranging from −2322 to −323 (Six genome sequence) was obtained from NCBI; (**B**) HEK293T cells were co-transfected with pRL-SV40 (renilla control) and pGL3-*Six2* promoter-LuC for 36 h. Luciferase activity was normalized to Renilla control. *p*-values were calculated by Student *t*-test. Values represents mean values ± SEM of triplicate experiments, *** *p* < 0.001 relative to control; (**C**) HEK293T cells were co-transfected with pRL-SV40 (renilla control) and pGL3-*Six2*-LuC for 36 h then were treated with LiCl of increasing dosages for 12 h. Luciferase activity was measured using dual luciferase reporter assay, normalized to Renilla control. Values were presented as mean ± SEM (*n* = 3), *** *p* < 0.001 relative to control; (**D**) mK3 cells were treated with LiCl of increasing dosages for 12 h. The *Six2* expression at protein level was tested by Western-blot. Values were presented as mean ± SEM (*n* = 3), ** *p* < 0.01 relative to control.

To further identify the relationship between *Six2* and LiCl, HEK293T cells were transfected with *Six2* promoter-LuC and treated with LiCl of increasing dosages. Then luciferase assay was performed to analyze the function of *Six2* promoter-LuC (Figure 3A,B). From the results of luciferase activity, we recognized that *Six2* promoter-LuC was functional and LiCl promoted *Six2* expression at low-concentration (0, 10, 20, 30, and 40 mM) and the promotion was inhibited it at high-concentration (50 mM) at mRNA level (Figure 3C) and protein level (Figure 3D). Moreover, this tendency was also confirmed in mK4 cells (Figure S1). Thus, we drew conclusions that LiCl may up-regulate *Six2* at low-concentration and down-regulate *Six2* at high-concentration at transcription and translation level.

### 2.3. LiCl Induces the Proliferation and Apoptosis of MM Cells with the Coordination of Six2

**Figure 4:**
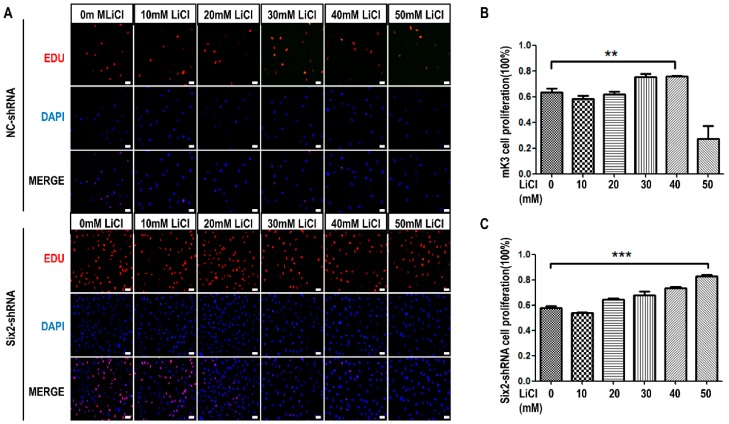

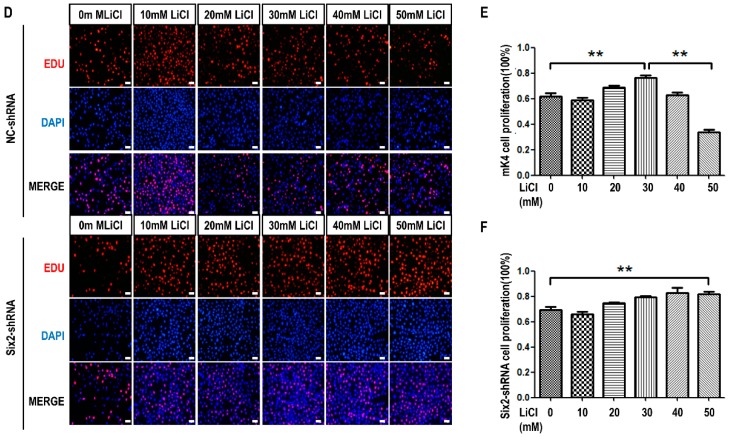
Knockdown of *Six2* gene inhibits cell proliferation while LiCl treatment of low-concentration promotes cell proliferation in mK3 and mK4 cells. (**A**) mK3 cells were transfected with negative shRNA control and *Six2*-shRNA for 36 h and treated with LiCl of increasing dosages for 12 h. Proliferating mK3 cells were labeled with EdU (red) and cell nucleus were stained with hoechst (blue). The EdU results were accessed by fluorescent microscope (200×) with the scale bar representing 20 μm and the respective pictures were merged to the purple one; (**B**,**C**) Statistical analysis of cell proliferation. Values were presented as mean ± SEM (*n* = 3), ** *p* < 0.01, *** *p* < 0.001 relative to control; (**D**) mK4 cells were transfected and were detected by EdU assay as same as mK3 cells in (**A**); (**E**,**F**) Statistical analysis of cell proliferation. Values were presented as mean ± SEM (*n* = 3), ** *p* < 0.01 relative to control.

To testify whether *Six2* is involved in the process of LiCl modulated cell proliferation, mK3 and mK4 cells were transfected with *Six2*-shRNA or negative control shRNA and treated with LiCl of increasing dosages. Firstly, we detected the efficiency of *Six2* deficiency by RT-PCR and Western blot, the results showed that the *Six2* expression is apparently decreased compared with negative shRNA controls, and 18S and β-tubulin, respectively, served as internal control of RT-PCR and Western blot (Figure 2A,B and Figure 3D). Moreover, we found that integral proliferation tendency was changed in mK3 cells transfected with *Six2*-shRNA. The proliferation was increased within groups treated with LiCl of increasing dosages (0, 10, 20, 30, 40, and 50 mM), compared with 0 mM LiCl treated cells though there was not significant difference between 0 and 10 mM (Figure 4A–C). This suggested that *Six2* deficiency inhibits the decreasing of mK3 cell proliferation induced by 50 mM LiCl. This result was independently repeated in mK4 cells (Figure 4D–F). In addition, to make the relationship between LiCl and *Six2* clearer, we detected the proliferation of *Six2*-overexpressed mK3 cells with LiCl treatment. The result showed that *Six2* promoted the increasing of cell proliferation induced by low LiCl concentration (10, 20, 30, and 40 mM) and maintained the decreasing of cell proliferation at high concentration (50 mM) (Figure S2A,B). The efficiency of *Six2* overexpression in mK3 cells was significantly checked by Western-blot (Figure S2C).

**Figure 5:**
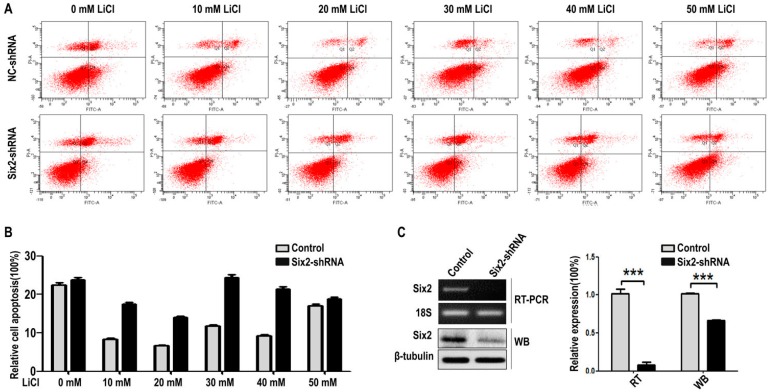
Knockdown of *Six2* gene accelerates cell apoptosis while LiCl treatment of low-concentration inhibits cell apoptosis in mK3 cells. (**A**) mK3 cells were transfected with negative shRNA control and *Six2*-shRNA for 36 h and treated with LiCl of increasing dosages. The apoptosis was detected by FCM; (**B**) Statistical analysis of cell apoptosis and histogram was drawn in GraphPad Prism 5; (**C**) The efficiency of knockdown *Six2* at mRNA and protein level, compared with internal control 18S and β-tubulin, respectively. Values were presented as mean ± SEM (*n* = 3), *** *p* < 0.001 relative to control.

Finally, we performed cell apoptosis assay of mK3 and mK4 cells using FCM. The results illustrated that LiCl inhibits mK3 cell apoptosis at low-concentration (10, 20, and 40 mM) compared with the non-LiCl control, and partially deletes this function at 30 and 50 mM, respectively, compared with 20 or 40 mM (Figure 5A,B). While *Six2* knock-down increased cell apoptosis rate in 10 mM to 50 mM LiCl treated mK3 cells, compared with the negative shRNA control cells (Figure 5A,B). Moreover, these findings were significantly repeated in another cell line, mK4 cells (Figure S3A,B). Then, the efficiency of *Six2* deletion was detected in mK3 and mK4 cells (Figure 5C and Figure S3C). These results suggested that *Six2* inhibits MM cell apoptosis and *Six2* plays a crucial role in the process that LiCl promotes cell proliferation and inhibit apoptosis at low-concentration.

## 3. Discussion

In this study, we carried out EdU assay to measure the proliferation of mK3 and mK4 cells treated with LiCl of increasing dosages to identify the cellular regulation of LiCl in mK3 cells proliferation. The results confirmed that LiCl promotes MM cell proliferation at low-concentration (0, 10, 20, 30, and 40 mM) and inhibits it at high-concentration (50 mM) (Figure 1A,B). To clarify that LiCl regulates *Six2* gene expression, we detected the mRNA expression of *Six2* gene and markers of *Wnt* and BMP signaling pathway. The results showed that LiCl increased the mRNA expression of *Six2* at the concentration of 10, 20, 30, and 40 mM compared with 0 mM but decreased it at 50 mM compared with the highest concentration (Figure 2A–D). Then, we further analyzed the relationship between LiCl and *Six2* by luciferase assay, discovering that the luciferase activity of *Six2* presented the same variation trend with *Six2* mRNA expression when concentration of LiCl rose (Figure 3B,C). Similarly, LiCl regulated the protein expression of *Six2* to the identical trend in mK3 cells (Figure 3D) and mK4 cells (Figure S1). Afterwards, to study whether *Six2* affects the function of LiCl to cell proliferation, we knocked down or overexpressed *Six2* and subsequently carried out EdU assay in mK3 cells. The result showed that the proliferation of mK3 cells increased as the concentration of LiCl rose from 10 to 50 mM, though *Six2* was silenced. It suggested that *Six2* deficiency inhibits the decreasing of mK3 cell proliferation induced by 50 mM LiCl (Figure 4A–C), which was notably confirmed in another cell line, mK4 cells (Figure 4D–F). Moreover, *Six2* promotes cell proliferation increasing induced by low LiCl concentration and convert the decreasing of cell proliferation induced by high LiCl concentration in mK3 cells (Figure S2). In addition, we also found that LiCl inhibits mK3 and mK4 cell apoptosis at low-concentration (0, 10, 20, 30, 40 mM) compared with the non-LiCl control, and partially deletes this function at high-concentration (50 mM) (Figure 5A,B and Figure S3). Meanwhile, *Six2* knock-down increased cell apoptosis rate in 10 M to 50 mM LiCl treated mK3 cells, compared with the negative shRNA control cells (Figure 5A,B).

LiCl has been reported to promote the proliferation of hippocampal neural stem/progenitor cells [13]. Here, we confirmed that LiCl also promoted MM cell proliferation at low-concentration (0, 10, 20, 30, and 40 mM). Differently, MM cell proliferation was decreased at high-concentration (50 mM) compared with the highest proliferation at 30 or 40 mM (Figure 1B,D). This variation trend was identical to the expression of *Six2* at mRNA level (Figure 2) and protein level (Figure 3C,D and Figure S1). As we all know, *Six2*, as a transcription factor, is an important gene involved in numerous signaling pathways and regulates organs development, and even impacts tumors generation [14]. It is reported that *Six2* is involved in the self-renewal of MM cells via maintaining cells at the progenitor state, which plays an essential role in kidney development [2]. Meanwhile, LiCl can inhibit cell proliferation [14]. These explained that there might exist an accumulation, which increased *Six2* expression as the concentration of LiCl rose in a low-concentration range (0, 10, 20, 30, and 40 mM) but the increasing was inhibited when the concentration of LiCl reached one limit (50 mM) [14].

In addition, *Six2* determines MM cell self-renewal associated with *Wnt* signaling pathway [6], a significant signaling pathway that functions in organs development and activated by LiCl [15]. Here we found the mRNA expression of *Wnt* signaling pathway markers in mK3 cells treated with LiCl of increasing concentration. The expression of *β-catenin* was increased gradually as concentration of LiCl rose (Figure 2B,D), corresponding to the cell proliferation promotion (Figure 1B,D); while the expression of GSK3β was decreased. Among BMP signal markers, the expression of BMP3 and BMP4 was increased, while the expression of BMP7 and BMPRII was reduced at 30 mM of LiCl concentration and then it was increased continuously, and the expression of BMPR-IA was reduced continually as concentration of LiCl increased (Figure 2B,D).

Moreover, the proliferation of mK3 cells was increased continuously as the concentration of LiCl increased though *Six2* was silenced (Figure 4B,C,E,F). These results may be caused by the interactions of *Six2* and other genes. Generally, *Six2* plays a leading role in the regulation of proliferation. As *Six2* was knocked down, other genes accumulate under the role of dose-increasing LiCl and play dominant roles in promoting proliferation [16,17,18]. Besides, we found that *Six2* deficiency increased the apoptosis of mK3 cells, compared with negative control cells at relative LiCl concentration (Figure 5A,B and Figure S3A,B). Furthermore, the abnormal rise of apoptosis at 30 mM of LiCl concentration implies that there may be another gene GSK3β that increased cell apoptosis. As concentration of LiCl rises, *Six2* is expressed increasingly while GSK3β is expressed decreasingly. Since *Six2* is knockdown, *Six2* expression activity is much less than GSK3β, so GSK3β plays a critical role in leading to increased apoptosis when the concentration of LiCl was 30 Mm [19]. However, *Six2* is expressed increasingly as concentration of LiCl rises, and partly inhibits the increasing apoptosis at 40 and 50 mM of LiCl concentration [20]. Surprisingly, there are some reports that claim the mammalian target of rapamycin (mTOR) pathway was involved in embryonic development; for instance, stimulation of the mTOR pathway with l-leucine rescued many developmental defects of establishment of sister chromatid cohesion *N*-acetyltransferase 2 (*Esco2*)-mutant embryos [21,22]. In addition, the reduction of mTOR pathway, combined with activation of canonical Wnt/β-catenin signaling, maintains human and mouse long-term Hematopoietic Stem Cells (HSCs) under cytokine-free conditions ex vivo, and this combining can increase the number of HSCs cells [23]. Therefore, these studies give us the idea that mTOR might be involved in the regulation of MM cell proliferation and apoptosis with the induction of LiCl and coordinating with some key factors of Wnt pathway, such as GSK3β.

In our research, we first studied significant function between *Six2* and LiCl in mK3 cells and recognized that *Six2* modulate the process that LiCl promote mK3 cell proliferation and inhibit the apoptosis. Furthermore, this regulation is crucial in MET to guarantee formation of nephron with complete function. We also discovered that there might be interactions between *Six2* and GSK3β that regulate cell apoptosis, which need further study.

## 4. Materials and Methods

### 4.1. Plasmids Construction

PGL3-*Six2* promoter-luciferase reporter gene were constructed and used in Dual-luciferase assays, relative to the internal control, plasmid pRL-SV40. The promoter of murine *Six2* was cloned from C57BL/6 mouse genomic DNA by PCR with the forward primer: CGTGCTAGCCCGGGCTATTTCCCAGGTCCCCTGGAATCCT and the reverse primer: CCGGAATGCCAAGCTCTTGCAGCTTTTTTAATAATATTAT. The PCR fragments were inserted into the XhoI/HindIII site (upstream of fly luciferase gene) of the pGL3-luciferase vector to create pGL3-*Six2* promoter-luciferase using the ligation-independent cloning. pGL3-basic vector (Promega, Madison, WI, USA) and pRL-SV40 was purchased from Promega. The pLKO.1-m.*Six2*-shRNA, *Six2* knockdown vector, the target sequence is CCTCCACAAGAATGAAAGCGT.

### 4.2. Cell Culture

mK3 (a mouse clonal cell line representing the un-differentiation stage of metanephric mesenchyme [24]) cells, mK4 cells (partial-differentiated MM cell line) and human embryonic kidney 293T (HEK293T) cells were cultured in Dulbecco’s modified Eagle’s medium (DMEM) (Gibico, Carls-bad, CA, USA), added 10% fetal bovine serum (FBS) (Gibico, Carlsbad, CA, USA) and penicillin (1000 units/mL) and streptomycin (1000 µg/mL) at 37 °C with 5% CO_2_, 100% humidity treated with LiCl of increasing dosages (0, 10, 20, 30, 40, and 50 mM).

### 4.3. Transfection and Luciferase Assays

HEK293T cells were incubated in 24-well plate (0.1 million each well) for 24 h and then transiently co-transfected with pGL3-*Six2* promoter-luciferase and plasmid pRL-SV40 utilizing Polyetherimide (PEI) (23966-2, polysciences, Warrington, PA, USA). mK3 cells were transfected with pLKO.1-m.*Six2*-shRNA using lentivirus vector. For luciferase assays, HEK293T cells were transfected with pGL3-*Six2* promoter-luciferase (500 ng/well) and pRL-SV40 (10 ng/well) for 36 h and then treated with LiCl of increasing dosages for 12 h. Luciferase (Luc) activity was assayed using Dual-Luciferase Reporter assay kit (Promega). Levels of firefly luciferase were standardized to those of Renilla.

### 4.4. Reverse Transcription-PCR (RT-PCR)

mK3 and mK4 cells were incubated for 24 h and treated by LiCl of increasing dosages for 12 h. The total RNA was isolated using Trizol (Invitrogen, Carlsbad, CA, USA). The *Wnt* and BMP signal markers, respectively APC primer F: TCTTCAGTGCCTCAACTTGC and primer R: GGAGACAGAATGGAGGTGCT, *β-catenin* primer F: GTCAGCTCGTGTCCTGTGAA and primer R: AGTGGCTGACAGCAGCTTTT, GSK3β primer F: ACTTCCTGTGGCCTGTCAGG and primer R: CAGCTTTTGGTAGCATGAAAGT, BMPR-IA primer F: ACATCAGATTACTGGGAGC and primer R: GCAAGGTATCCTCTGGTGCT, BMPRII primers F: CTTTACTGAGAACTTTCCAC and primer R: CCAAAACATAAGGCGACTATC, BMP3 primer F: TGGCTCTATGACAGGTACAG and primer R: ATGTTCTCCGACTTGGTTAG, BMP4 primers F: TTGTTCAAGATTGGCT CCCAAG and primer R: GGCATAATAAAACGACCATCAGC, BMP7 primers F: ACCCTTCATGGTGGCCTTCT and primer R: CCTCAGGGCCTCTTGGTTCT, and *Six2* primers F: GCCAAGGAAAGGGAGAACAG and R: TGAGCAACAGAGCGGGACT were detected by RT-PCR and the expression activities of these markers were normalized to internal control (18 s). The relative gene expression was analyzed using Image J Software (National Institutes of Health, Bethesda, MD, USA).

### 4.5. Western Blot

mK3 and mK4 cells were cultured in 6-well plates for 24 h and treated with LiCl of increasing dosages for 12 h. Furthermore, the Western blot assays was processed with antibodies *Six2* (1:600, proteintech, Chicago, IL, USA) and internal control β-tubulin (1:5000, proteintech) followed by the research “Identification of a thyroid microsomal antigen by Western blot and immune-precipitation” [25].

### 4.6. 5-Ethynyl-20-deoxyuridine (EdU) Assays

mK3 and mK4 cells were incubated in 24-well plate (0.05 million/well) for 24 h and treated with LiCl of increasing dosages for 12 h. Furthermore, the proliferation of mK3 cells were determined in vitro via the EdU DNA Proliferation in Detection kit (RiboBio, Guangzhou, China).

### 4.7. Flow Cytometry Apoptosis Assays

mK3 and mK4 cells were treated with LiCl of increasing dosages for 12 h and the apoptosis assays were measured by flow cytometers (FCM) with Annexin V-FITC Apoptosis Detection Kit (KeyGEN BioTECH, Nanjing, China).

### 4.8. Statistical Analysis

All of the work was performed in triplicate, and the results are presented as the mean ± standard error of the mean (SEM). We used the GraphPad Prism 5 software (GraphPad, San Diego, CA, USA) to calculate the statistical results. *p*-values were counted by Student’s *t*-test and * *p* < 0.05, ** *p* < 0.01, and *** *p* < 0.001 were considered statistical differences.

## 5. Conclusions

Our study implies that LiCl can induce the proliferation and apoptosis of MM cells coordinating with *Six2*.

## Supplementary Materials

Supplementary materials can be found at www.mdpi.com/1422-0067/17/9/1504/s1.

## Abbreviations

MM	metanephric mesenchymal
UB	ureteric bud
*Six2*	Sine oculis homeobox homolog 2
LiCl	lithium chloride
BMP	bone morphogenetic protein
FITC	Fluorescein isothiocyanate fitc
HEK	human embryonic kidney
MET	mesenchymal-epithelial-transition
DMEM	Dulbecco’s modified Eagle’s medium
