# Supplementary material for: Six2 Is a Coordinator of LiCl-Induced Cell Proliferation and Apoptosis
Source: Int J Mol Sci. 2016 Sep 8;17(9):1504. doi: 10.3390/ijms17091504 (PMC5037781; doi:10.3390/ijms17091504)
Supplement: Supplementary file 1 [file ijms-17-01504-s001.pdf]

# Supplementary Materials: *Six2* Is a Coordinator of LiCl-Induced Cell Proliferation and Apoptosis

**Pan Ju, Yuru Zhou, Ya Zhao, Yajun Xie, Yaoshui Long, Yuping Gu, Dongsheng Ni, Jianing Liu, Zhongshi Lyv, Zhaomin Mao, Jin Hao, Yiman Li, Qianya Wan, Quist Kanyomse, Yamin Liu, Yue Xiang, Ruoli Wang, Xiangling Chen, Junman Zhang, Xihan Liu, Hui Zhao, Qin Zhou and Ge Li**

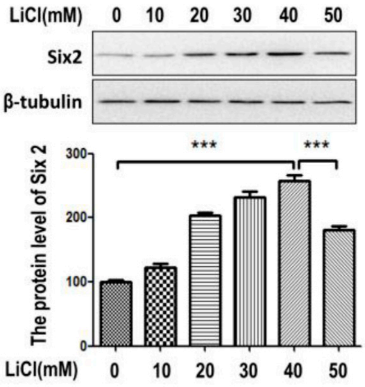


**Figure S1.** LiCl regulates the expression of Six2 at protein level in mK4 cells. mK4 cells were treated with LiCl of increasing dosages for 12 h. The Six2 expression at protein level was tested by Western-blot. Values were presented as mean ± SEM ($n = 3$), *** $p < 0.001$ relative to control.

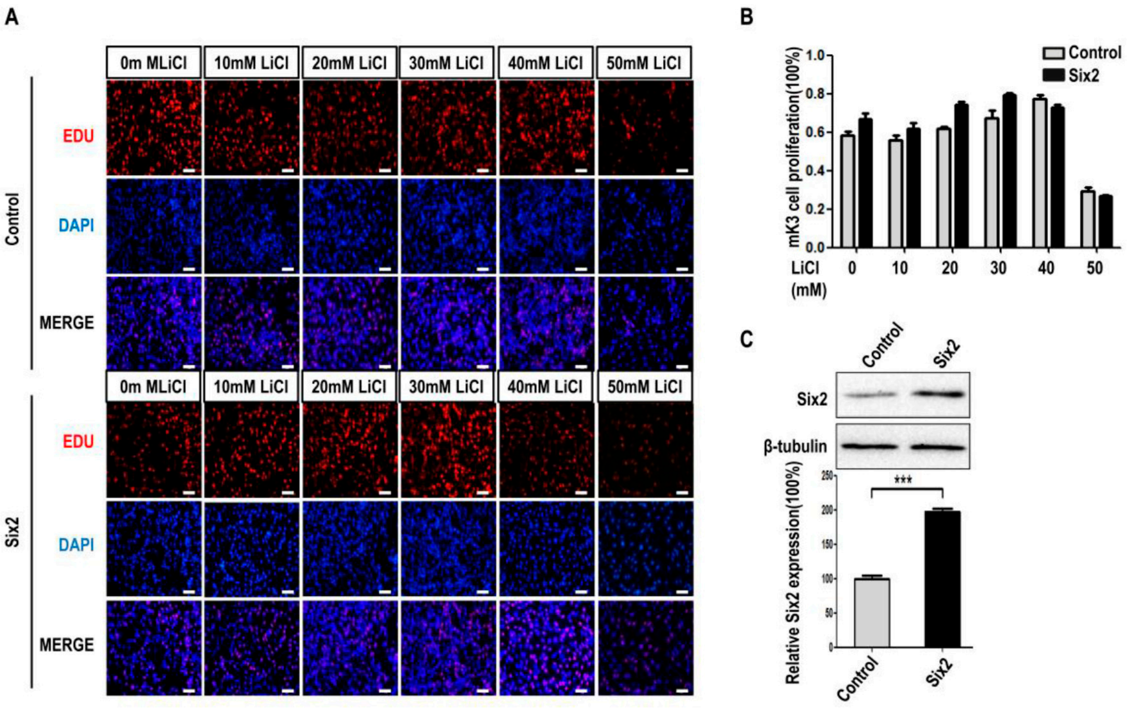


**Figure S2.** Overexpression of *Six2* gene promotes cell proliferation induced by low-concentration of LiCl. (**A**) mK3 cells were transfected with *Six2* overexpression vector and control vector for 36 h and treated with LiCl of increasing dosages for 12 h. Proliferating mK3 cells were labeled with EdU (red) and cell nucleus were stained with hoechst (blue). The EdU results were accessed by fluorescent microscope (200×) with the scale bar representing 20 μm and the respective pictures were merged to the purple one; (**B**,**C**) Statistical analysis of cell proliferation. Values were presented as mean ± SEM ($n = 3$), *** $p < 0.001$ relative to control.

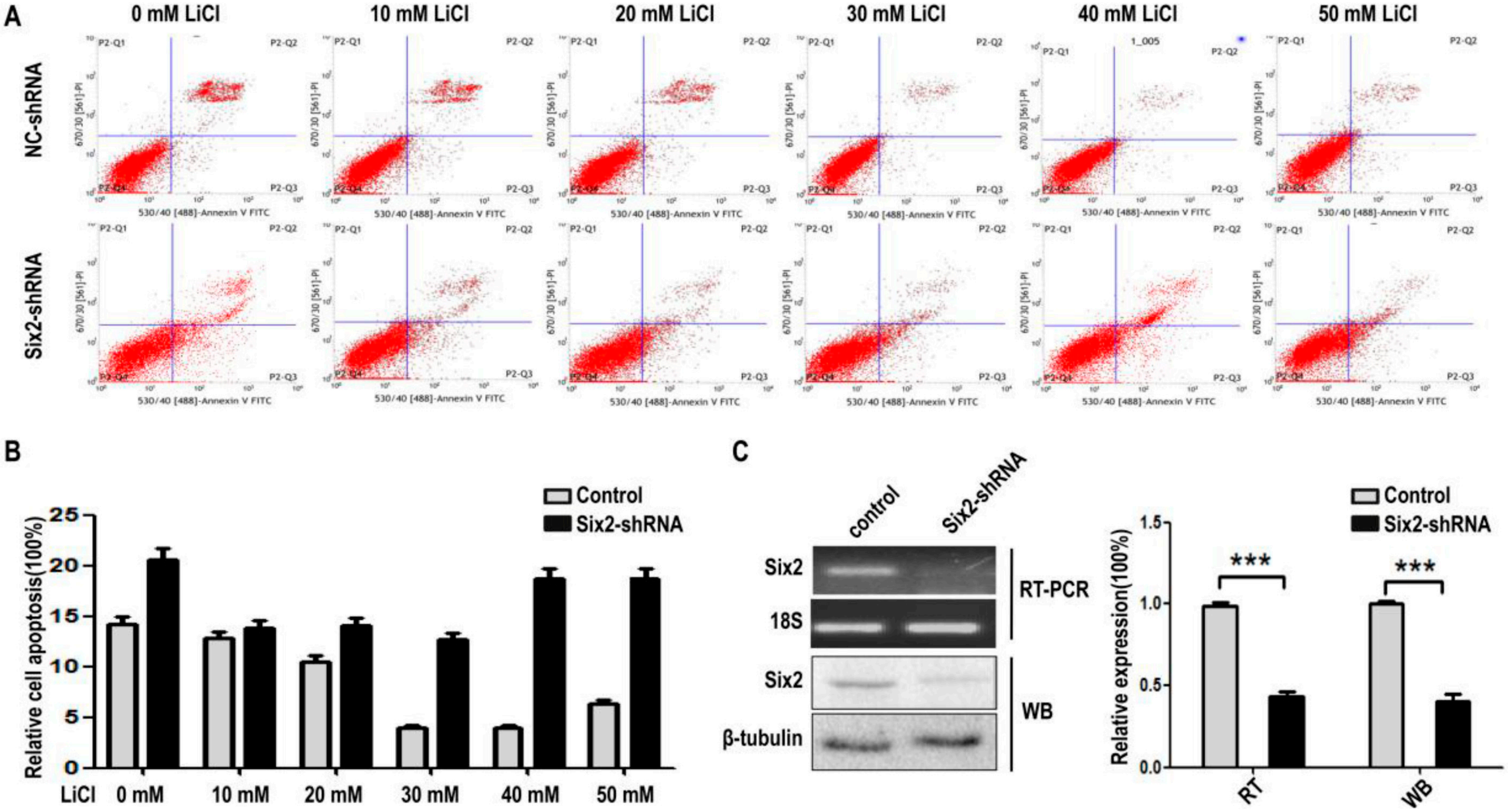


**Figure S3.** Knockdown of *Six2* gene accelerates cell apoptosis while LiCl treatment of low-concentration inhibits cell apoptosis in mK4 cells. (**A**) mK4 cells were transfected with negative shRNA control and Six2-shRNA for 36 h and treated with LiCl of increasing dosages. The apoptosis was detected by FCM; (**B**) Statistical analysis of cell apoptosis and histogram was drew by GraphPad Prism 5; (**C**) The efficiency of knockdown Six2 at mRNA and protein level, compared with internal control 18S and β-tubulin respectively. Values were presented as mean ± SEM ($n = 3$), *** $p < 0.001$ relative to control.
